# Comprehensive Review Tapered Optical Fiber Configurations for Sensing Application: Trend and Challenges

**DOI:** 10.3390/bios11080253

**Published:** 2021-07-27

**Authors:** Bakr Ahmed Taha, Norazida Ali, Nurfarhana Mohamad Sapiee, Mahmoud Muhanad Fadhel, Ros Maria Mat Yeh, Nur Nadia Bachok, Yousif Al Mashhadany, Norhana Arsad

**Affiliations:** 1 Department of Electrical, Electronic and Systems Engineering, Faculty of Engineering and Built Environment, Universiti Kebangsaan Malaysia, UKM, Bangi 43600, Malaysia; p103537@siswa.ukm.edu.my (B.A.T.); p96194@siswa.ukm.edu.my (N.A.); p103517@siswa.ukm.edu.my (N.M.S.); p97209@siswa.ukm.edu.my (M.M.F.); p100898@siswa.ukm.edu.my (R.M.M.Y.); p110441@siswa.ukm.edu.my (N.N.B.); 2 Department of Electrical Engineering, College of Engineering, University of Anbar, Ramadi 00964, Anbar, Iraq; yousif.mohammed@uoanbar.edu.iq

**Keywords:** tapered optical fiber, optical sensors, Bragg grating, microstructure, refractive index sensor

## Abstract

Understanding environmental information is necessary for functions correlated with human activities to improve healthcare quality and reduce ecological risk. Tapered optical fibers reduce some limitations of such devices and can be considerably more responsive to fluorescence and absorption properties changes. Data have been collected from reliable sources such as Science Direct, IEEE Xplore, Scopus, Web of Science, PubMed, and Google Scholar. In this narrative review, we have summarized and analyzed eight classes of tapered-fiber forms: fiber Bragg grating (FBG), long-period fiber grating (LPFG), Mach–Zehnder interferometer (MZI), photonic crystals fiber (PCF), surface plasmonic resonance (SPR), multi-taper devices, fiber loop ring-down technology, and optical tweezers. We evaluated many issues to make an informed judgement about the viability of employing the best of these methods in optical sensors. The analysis of performance for tapered optical fibers depends on four mean parameters: taper length, sensitivity, wavelength scale, and waist diameter. Finally, we assess the most potent strategy that has the potential for medical and environmental applications.

## 1. Introduction

Understanding environmental information is necessary for the correct functioning of processes associated with individual actions to improve indoor and outdoor conditions [1]. However, it needs monitoring and management of essential environmental factors that may be classified into physical parameters such as temperature, pressure, humidity, and biological characteristics, such as concentration of contaminants and bio-hazards, and pathogens, such as viruses [2]. Over the years, optical sensors have become an attractive topic for researchers in many applications due to their benefits such as compact size, low weight, electromagnetic protection, high stability, and the ability to study sensor arrays remotely. Fiber Bragg grating (FBG) devices are based on a diffraction grating. The concept of grating refers to periodic core refractive index change. When light passes through the grating structure, it reflects a portion of each grating layer [3,4,5]. Long-period fiber grating (LPFG) has periods ranging from hundreds of microns to millimeters, linking incoming light led by a propagation direction in the core to various forward-propagating cladding modes of high diffraction order m in an optical fiber, which fade fast due to scattering losses [6,7,8]. A Mach–Zehnder interferometer (MZI) measures the relative phase shift changes between two beams formed by dividing light from a single source. Many applications were covered, such as couplers, ring resonators, splitters, tapers, gates, and terminators [9,10]. A photonic crystal fiber (PCF) is a periodical optical nanostructure that controls photon mobility [11]. Surface plasmon resonance (SPR) SPR is the resonant vibration of charged electrons at the interface of negative and positive permeability material by generated light to detect material absorption on planar metal (commonly gold/silver) surfaces [12,13]. Multi-taper devices are integrated by two types of fiber (single-mode /multi-mode) [14]. Optical fiber loop ring-down (FLRD) links two optical couplers into the optical ring [15,16]. Optical tweezers are formerly known as single-beam gradient force traps that capture and manipulate tiny particles using a highly powerful laser beam [17,18].

## 2. Background

In this part, we will provide a selection of current literature covering a wide range of research techniques and biological systems. In the 1960s, many studies have attempted to develop optical fiber in different fields such as communication, medicine, the environment, and many others. Essentially, optical sensing originated from fiber communication. The employment of lasers with optical fiber enables accurate and sensitive detection compared with other techniques. This method has the advantage of notable features such as interference resistance, adaptability, real-time parameter monitoring, and small size [19,20,21,22,23]. Many optical fiber structures use lasers such as FBG, fiber-optic interferometer, optical fiber MZI, buffer structure, Fabry–Perot, directional couplers, and optical fiber waveguides to a biosensor [24,25,26,27,28]. Tapered fiber production has been shown to employ several processes, including laser ablation, electron beam lithography, vapor–liquid–solid operations, and fiber pulling. Among these processes, flame heating is one of the most adaptable to produce tapered fibers with good physical properties [29,30,31]. Optical sensors can detect changes in optics properties such as refractive index (RI), absorption, reflection, and fluorescence related to the physical parameters of the investigated environment such as pressure, strain, temperature, and chemical composition. In contrast, it is simple to create optical sensors by guiding light to and collecting light from the measuring location, a process known as extrinsic sensing [32], or by using the fiber itself as the transducer, a technique known as intrinsic sensing [33]. Assessment approaches based on optical fibers received considerable attention in various analytical fields, including chemical and biological sensing, environmental and structural health monitoring, and medical diagnostics. Their vast range of designs and methods enable optical fibers to construct sensitive and selective sensors for real-world circumstances [34,35,36]. The tapered fiber technique is one of the most widely used in the production of optical sensor components. The tapered fiber technique uses the evanescent wave (EW) tapered-propagating mode to determine RI or chemical composition properties [37]. Recently, tapered optical fibers have been used to create sensors including polarizers, sub-micron wire, light amplification, and near-field microscopy [38,39,40,41,42]. As shown in the literature, the shape of the tapered fiber is critical to its sensor function, with lower diameter taper waists giving increased sensitivity [43,44]. Additionally, tapered fiber optics may be used as light probes to evaluate characteristics of interest with spatial precision on the scale of micrometers, allowing direct examination of biological samples at a cellular level [45]. A study shows the relationship between the integrated photon intensity with particle size and tip radius by scanning tunneling microscopy. The isochromatic photon map varies with the wavelength and geometric asymmetry of the STM tip [46]. In recent years, optical fiber tweezers have garnered significant attention in optical trapping because of their ease of manipulation and design that is small and easy to fabricate [47]. A study shows the use of spectral-width tapered FBG in microsurgical force sensors [48]. Label-free sensors use taper fiber and FBG to identify various cancers [49].

## 3. Overview of Tapered Fiber

**Figure 1:**
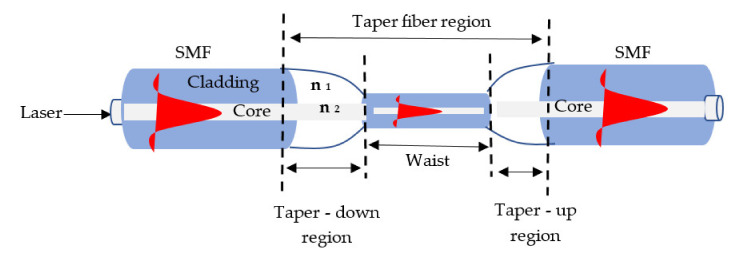
Schematic illustration of the tapered fiber standard [56].

Optical fiber is divided into two types depending on the number of modes (single mode fiber (SMF) and multi-mode fiber (MMF)) and refractive index (RI). In SMF, only one kind of light beam may propagate through the fiber core with a diameter of 5–10 μm and 125 μm of cladding mode. In MMF, multiple light beam modes can propagate in the fiber core with diameter (50–100) μm and 125 μm of cladding mode [50,51]. In contrast, tapered fibers are created by stretching a conventional SMF to generate a reduced core diameter shape at the lowest diameter named as the waist. In addition, the waist is a transition zone whose cladding and core diameters decrease when the SMF rated size decreases to the order of micrometer or even nanoscale. The field distribution shifts as the wave propagates through the transition zones due to the change in core/cladding diameters along the path. Thus, energy transfers change with the rate of diameter change, resulting in the loss of propagating wave power and increase loss with the built tapered fiber [52,53]. Flame heating techniques and lasers are used to generate tapered optical fibers. Besides, the tapered length, waist diameter, and refractive index (RI) parameters have been influential in optical properties [54]. Fiber tapers are classified into two types, adiabatic and non-adiabatic. In adiabatic designs, the angle of the taper transition area is a modest $10^{-4}$–$10^{-3}$ rad and the optical fiber cylinder symmetric is maintained (with a taper diameter ratio to the core beginning diameter $a/a_{0}$ between 0.2 and 1), resulting in most optical power remaining in the basic mode. The non-adiabatic taper, on the other hand, has a larger taper angle [55,56]. Standard tapered fiber schematic depiction is shown in Figure 1.

## 4. Taxonomy of Literature Review on Tapered Optic Fiber

**Figure 2:**
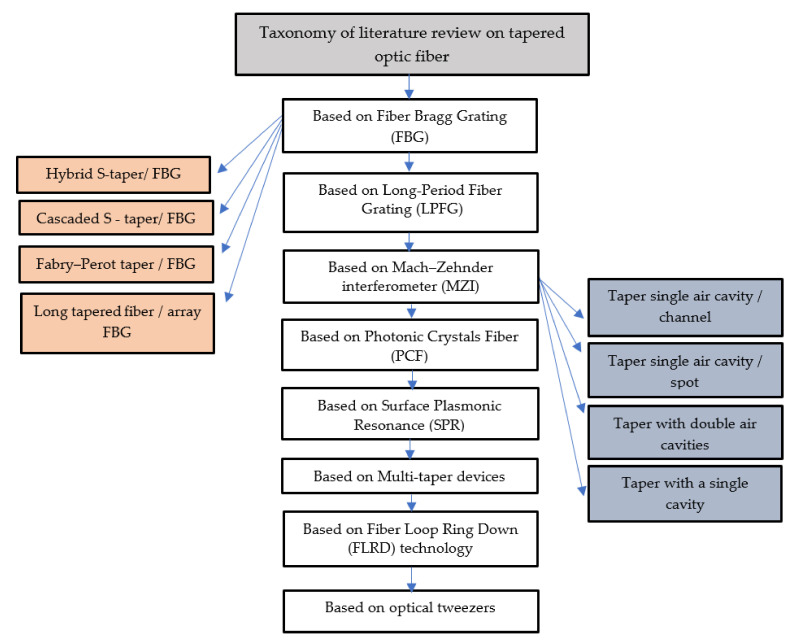
Taxonomy of literature research on tapered optic fiber.

Our review selected, evaluated, and analyzed (22) empirical research on optical sensors based on tapered optic fiber, as shown in Table 1. We extract relevant literature from academic databases, such as Science Direct, which provides diverse scientific research across all fields; Scopus, which provides extensive coverage of work from all disciplines; Web of Science, which demonstrates a comprehensive range of various topics in all literature; IEEE, which is recognized as scientifically accurate and pro-science. The section that follows offers a taxonomy of tapered fiber structures and a literature review, as shown in Figure 2.

### 4.1. Based on Fiber Bragg Grating (FBG)

**Figure 3:**
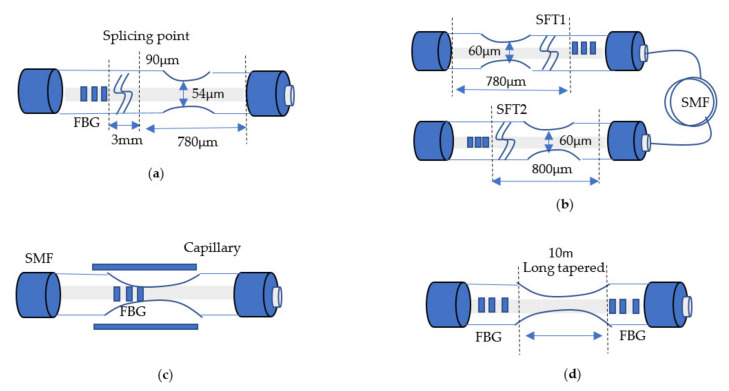
Tapered fiber optic based on FBG structures. (**a**) Illustration of hybrid S-taper/FBG structure [62]. (**b**) Illustration of cascaded S-taper/FBG structure [63]. (**c**) Illustration of Fabry–Perot/tapered FBG structure [70]. (**d**) Illustration of long tapered fiber/array of FBG structure [71,72].

FBG techniques are widely employed in photonics sensors devices, having many forms: uniform, chirped, and superstructure [57,58], and some research investigated laser fiber cavities and optical processors to manage complicated optical signals using the FBG technique [59,60,61]. Figure 3 shows tapered fiber based on FBG structures.

#### 4.1.1. Hybrid S-Taper/FBG Structure

A study has proposed a temperature-compensated fiber laser refractometer based on a compact hybrid S fiber taper (SFT)/FBG form. SFT assists as both a sensor and a band-pass filter (BPF) to customize the FBG reflection, a microsize of 0.78 mm. The surrounding refractive index can control the erbium-doped fiber ring laser (FRL) output power due to the RI-dependence intra-cavity loss (SRI). The refractometer has a high sensitivity of 269.76 dB/RIU across the measuring range of 1.3330–1.4060 [62]. Because of the big bending radius, the cladding layer is activated at the initial bending region, as light propagates in the SFT; at the second bending area, the stimulated cladding modes are partially back into the core. The transmission spectrum is described by Equation (1) [62].

(1) $$I=I_{cor\;}+I_{cla}+2\;\sqrt{I_{cor}\cdot \;I_{cla}\;}\cos(2\pi \;\Delta n_{eff}\;\cdot \;L_{eff}\;/\lambda )$$

where $I_{cor}$ and $I_{cla}$ indicate the intensity of the core and cladding modes, respectively, $n_{eff}$ denotes the effective RI difference between the core and cladding modes, $L_{eff}$ denotes the interferometer effective length, and $2\pi \;\Delta n_{eff}$ is the phase deviation between the core and cladding.

#### 4.1.2. Cascaded S Fiber Taper/FBG Structures

A dual-point fiber sensor for RI characteristics uses a tiny S fiber taper cascaded (SFTC) with Bragg grating suggested. It achieved the multiplexing capability of the sensor by cascading. The RI sensitivities range was 1.3540–1.3810, and sensing point 1 and sensing point 2 reached 459.974 nm/RIU and 420.781 nm/RIU, respectively. RI sensor benefits from a small sensing zone, a well-defined interference pattern, and a high RI sensitivity, allowing it to be utilized as a low-cost RI sensor with multiplexing capabilities. This technique shows the benefits of compact size, simple construction, and high accuracy [63]. An optical sensor that uses dual parameters to detect temperature and pressure was reported. The two major sensing structures are a cascaded Bragg fiber grating and a ball-shaped extrinsic Fabry–Perot interferometer. The taper is produced by extending it under the effect of a fusion-splicer arc discharge. Additionally, use the femtosecond laser to make a point-by-point direct method and a Bragg grating in the taper region. The results were in the temperature range of 40 to 400 °C and 0 to 2 MPa [64]. The Bragg phase-matching condition may characterize the connection between the shape of the grating and its reflection properties, as shown in Equation (2) [64].

(2) $$\lambda_{B}=2n_{eff}\cdot \Lambda$$

where $\lambda_{B}$ denotes the Bragg resonance wavelength, $n_{eff}$ denotes the fiber core effective index, and Λ denotes the grating pitch, and the phase difference in the two reflected laser beams is given in Equation (3).

(3) $$\frac{4\pi nL}{\lambda_{m}}+\phi_{0}=\left(2m+1\right)\pi$$

where m is a positive integer, $\lambda_{m}$ is the wavelength of the order interference dip, and spectrum fringe spacing can be calculated in Equation (4).

(4) $$\Lambda_{FP}=\frac{\lambda^{2}}{2nL}$$

#### 4.1.3. Fabry–Perot/Tapered FBG

According to a literature survey, Fabry–Perot interferometers (FPIs) have several essential properties such as low cost, high resolution, electro-magnetic interference avoidance, compact, and reliability [65,66], and they are widely used for various sensing measurements, including temperature, strain, and gas pressure [67,68]. A study shows a strain sensing FP sensor system based on tapered FBG. The fiber taper into the capillary with FBG was employed [69,70]. A new in-line FP strain sensor based on a tapered FBG-in-capillary construction to detect temperature was proposed. It is created by inserting a fiber taper containing FBG into a capillary, resulting in an ultra-long active length and an ultra-short interference length and a strain sensitivity of 1129.44 pm/με. The intensity of reflection of FP can be calculated by Equation (5) [70].

(5) $$I=I_{1}+I_{2}\;2\sqrt{I_{1}-I_{2}}\;\cos\left(\frac{4\pi \cdot n_{air}\cdot \;L_{FP}}{\lambda }\;\phi_{0}\right)$$

where $I_{1}$ and $I_{2}$ are the intensity of two reflection rays, $n_{air}$ is the air refractive index, λ is the light wavelength, and $\phi_{0}$ is initial phase difference.

(6) $$\lambda_{m}=\frac{2n_{air}\;\cdot \;L_{FP}}{m+1/2}$$

Assuming, $\phi_{0}=0$, when $4\pi \cdot n_{air}\cdot \frac{L_{FP}}{\lambda }=\left(2m+1\right)\pi$, m (an integer) $\lambda_{m}$ is the wavelength of order resonance dip as shown above in Equation (6) [70]. The free spectral range (FSR) of the interference fringe of the FPI can be calculated using Equation (7) [70].

(7) $$FSR=\lambda_{dip}\left(m\right)-\lambda_{dip\;}\left(m-1\right)\;\approx \;\frac{\lambda^{2}}{2n_{air}\;L_{FP}}$$

#### 4.1.4. Long Tapered Fiber/Array of FBG

Insights drawn from scholarly literature are supplemented with the FBG tapered techniques. Typically, long tapered fiber with an array of FBG is based on altering the fiber diameter through manufacturing. An array FBG was inscribed during the drafting process to create a tapered optical fiber with an adiabatic transition from single mode to become a few-mode part. The reflected signal from the tapered fiber has a spectral width of 4 nm. At some wavelengths, the reflected signal intensity might reach up to 5%. [71]. The long tapered fiber method based on diameter was altered by a factor of 1.5, which was severely constrained by weakening the optical fiber light-guiding characteristics with decreasing frequency value [72]. However, increasing fiber diameter can be considered without affecting fiber wave guiding characteristics, but it results in a few-mode propagation regime and signals quality degradation. FBG reflects light at a specific wavelength and broadcasts light at all other wavelengths due to the refractive index variation caused by the exposure of the fiber core to ultraviolet radiation.

### 4.2. Based on Long-Period Fiber Grating (LPFG)

**Figure 4:**
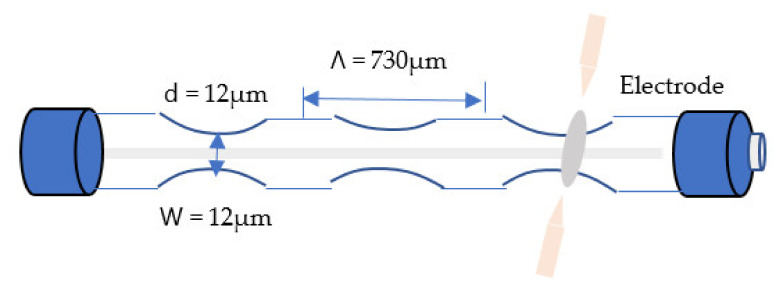
Tapered fiber optic based on long-period fiber grating (LPFG) structure [75].

A fiber sensor based on the LPG method was presented in other studies, which utilized a tapered fiber. It transfers light coupled to cladding modes to a biconically optical taper across a liquid phase gap LPG. This type of structure allows for the monitoring of the LPG characteristics employed in devices or systems and the construction of some specific devices [73,74]. The authors present tapered fiber optic sensors based on LPG for detection in chemistry and biology. The sensor is built using a low-cost arc heating method to inscribe periodic microtapers in a single fiber optic. SMF periodic tapering allows for coupling of the fundamental core mode to the cladding mode generated by a transmission spectrum resonant frequency. The resonant wavelength varies linearly as the refractive index changes. Experimentally established refractive index sensitivity of 8188 nm/RIU, which is substantially higher than previously published RI sensitivity values for LPG sensors. However, the sensitivity is controlled by periodic tapers, grating length, and waist diameter [75,76]. A periodic tapered LPFG is a selection-based filter with a transmission spectrum similar to a conventional LPFG but with several resonances caused by coupling between the main guided mode $LP_{o1},\;LP_{or}$ cladding modes resonance wavelengths as shown in Equation (8) [77].

(8) $$\lambda_{r}=(n_{eff}^{cor}-n_{eff}^{cla,r})\Lambda /q$$

where, Λ is the period of the grating, $\lambda_{r}$ is the resonance wavelength, $n_{eff}^{cor}$ and $n_{eff}^{cla,r}$ denote the effective refractive, r is order cladding mode, respectively, and q denotes order of diffraction. Consequently, the resonance wavelength coupled the guided mode to the cladding mode with the narrow transmission spectrum due to the high attenuation cladding layer. In refractive index sensing, the relationship between the resonant wavelengths and the RI of the external medium is represented in Equation (9) [78]

(9) $$\frac{d\lambda_{r}}{dn_{ext}}\;\lambda_{r}\;\delta \varepsilon_{ext}$$

where δ is the overall sensitivity factor, and $\varepsilon_{ext}$ is the LPFG response coefficient to fluctuation in the external RI. The glass is crushed axially by applying several arc discharges throughout the length of the fiber [79]. However, the periodic changes in the diameter result in a tapered LPFG. The periodical taper area generates evanescent waves, and lights leak out. The effective eigenmode RI would be considerably altered. Figure 4 shows tapered fiber based on long-period fiber grating (LPFG) structure.

### 4.3. Based on Mach–Zehnder Interferometer (MZI)

**Figure 5:**
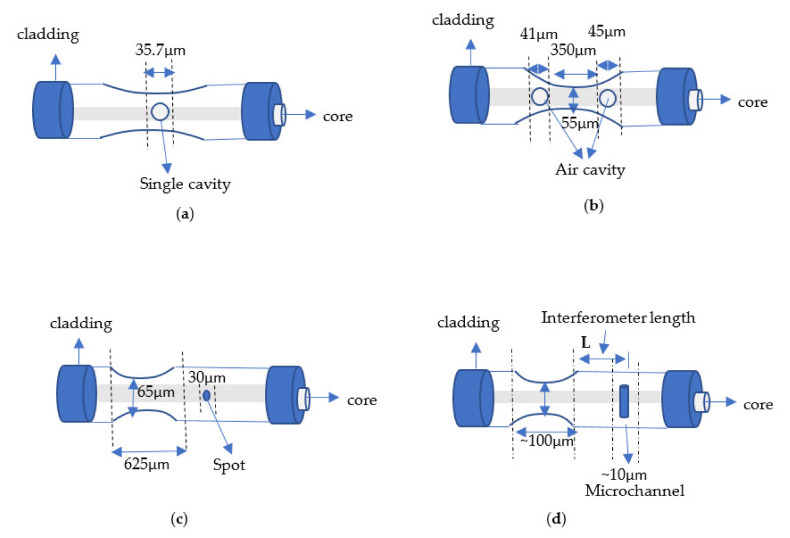
Tapered fiber based on Mach–Zehnder (MZI) structures. (**a**) Illustration of tapered based on single cavity [82]. (**b**) Illustration of tapered based on double air cavities [87]. (**c**) Illustration of tapered based on air cavity and spot [90]. (**d**) Illustration tapered based on single air cavity/channel [91].

The MZI method is a sort of interferometer dividing the emitting light beam from one source to two collimated beams. MZI can monitor optical path changes between light/medium after splitting and a 3 dB splitter is required. In addition, the two split lights only cross MZI. Furthermore, MZI has been widely used in optical fiber sensing technology, and several methods in modern years have been suggested [80,81,82,83]. Figure 5 shows tapered fiber optic based on Mach–Zehnder (MZI) structures.

#### 4.3.1. Taper with a Single Cavity

The tip of the optical sensor is manufactured using the following ways: femtosecond laser, fusion splicing, and heating and pulling processes [84]. An ultra-compact microfiber sensor head for high-temperature detection is presented. The method was a Mach–Zehnder interferometer based on an inner air cavity. The refractive index sensitivity was 4202 nm/RI [85]. The sensitivity of stress can be defined as shown in Equation (10), to measure temperature, RI, and strain, where *m* is an integer, *L* is the cavity length, λ is the wavelength, $\Delta n_{eff}^{clad}$, $\Delta n_{eff}^{air}$ is the RI difference between the core/air-cavity mode, and ε is the strain [82,86].

(10) $$\frac{\Delta \lambda_{dip}}{\Delta \varepsilon }=\frac{2}{2m+1}\left[\Delta n_{eff}\frac{\Delta L}{\Delta \varepsilon }+L(\frac{\Delta n_{eff}^{clad}}{\Delta \varepsilon }-\frac{\Delta n_{eff}^{air}}{\Delta \varepsilon })\right]\varepsilon =\frac{\Delta L}{L}$$

Because of this equation’s hypothesis $\left[\begin{array}{c} \Delta \lambda_{1}\\ \Delta \lambda_{2} \end{array}\right]=\left[\begin{array}{cc} KT_{1} & Kn_{1}\\ KT_{2} & Kn_{2} \end{array}\right]\left[\begin{array}{c} \Delta T\\ \Delta n \end{array}\right]$, the dipping wavelength change occurs as a result of temperature, and strain change is $\left[\begin{array}{c} \Delta \lambda_{1}\\ \Delta \lambda_{2} \end{array}\right]=\left[\begin{array}{cc} KT_{1} & K\varepsilon_{1}\\ KT_{2} & K\varepsilon_{2} \end{array}\right]\left[\begin{array}{c} \Delta T\\ \Delta \varepsilon \end{array}\right]$, ref. [84].

#### 4.3.2. Taper with Double Air Cavities

In this part, the technique concept uses two single-mode fiber air cavities (SMF) produced by femtosecond laser, fusion-splicing, similar to the preceding structures and somewhat tapered methods. A novel tapered fiber method based on fiber MZI with a pair of inner air bubbles is reported. The technique included exposure to the core end face of SMF using femtosecond laser pulses with power calibrated to 1 mW. The results were a nonlinear wavelength shift of 1485 nm near to where the dip was located and a sensitivity of 28 nm/vol [87]. The previous hypothesis presupposes that the length of the exterior cavity is constant regardless of temperature changes. However, the interior air cavity is very tiny, and the fluctuation in it caused by the material’s thermal expansion is negligible [88]. The sensitivity can be expressed by Equation (11) [88].

(11) $$\frac{d\lambda_{v}}{dn_{ext}}=-\frac{2L}{2K+1}\;\frac{\partial_{cl,m}^{eff}}{\partial n_{ext}}/\left[1-\frac{2L}{2K+1}\;\left(\;\frac{\partial_{core}^{eff}}{\partial \lambda }-\frac{\partial_{cl,m}^{eff}}{\partial \lambda }\;\right)\right]$$

where  L is the interferometer length, $\partial_{core}^{eff}$ and $\partial_{cl,m}^{eff}$ are the effective refractive indices, $n_{ext}$ is the external refractive index, k is an integer, and $\lambda_{v}\;$ is the maximum attenuation wavelength. In the external medium, the optical power is associated with the wave evanescent of the cladding. Consequently, sensitivity is increased by decreasing the fiber diameter of the sensor’s contact area with the external medium.

#### 4.3.3. Taper with Single Air Cavity/Spot

Several studies have explored the effects of optical taper-based MZI on combining micro holes or microfluidic channels processed by femtosecond laser. The core is combined with higher-order cladding during a single taper exists to narrow down the optical fiber [89]. The MZI is proposed based on a micro spot (microcavity) at a specific distance from the tapered sensor, which is used as a temperature sensor. The results were the refractive index sensitivities of (213.235 and 215.294) nm/RIU [90]. The relationship between the fiber Mach–Zehnder interferometers (FMZI) peak attenuation wavelength of $\delta \lambda_{m,RI\;}$, and the refractive index (RI) was investigated, as shown in Equation (12), while the relationship of attenuation peak wavelength $\delta \lambda_{m,T\;},\;$ and temperature can be expressed as shown in Equation (13) [90]. Moreover, the temperature increase leads to the shifting of the attenuation peak wavelength to achieve a perfect refractive index by considering the temperature impact; the variations in temperature interference orders’ peak wavelengths attenuation depend on the inherent cross-sensitivity effects FMZI between the RI and temperature.

(12) $$\delta \lambda_{m,RI\;}=\frac{2\left(\;\Delta n_{eff,RI\;}-\delta \lambda_{eff,RI\;}\right)L}{2m+1}-\frac{2\;\Delta n_{eff,RI\;}L}{2m+1}=\frac{-2\delta \lambda_{eff,RI\;}L}{2m+1}$$

(13) $$\delta \lambda_{m,T\;}=\frac{2\left(\;\Delta n_{eff,T\;}-\delta \lambda_{eff,T\;}\right)L}{2m+1}-\frac{2\;\Delta n_{eff,T\;}L}{2m+1}=\frac{\;2\delta \lambda_{eff,T\;}L}{2m+1}$$

#### 4.3.4. Taper with Single Air Cavity/Channel

Microchannel structure can work as an interferometer method through optical fiber as the spot and a channel for fluids. Yingyu et al. discovered MZI with taper and microchannel. The method used the integration of femtosecond laser with arc fusion splicing for fabrication. The results were sensitivity of ~361.29 nm/RIU and RI range from 1.33 to 1.35 [91]. The sensitivity can be enhance by using microchannels/spots (holes) and air cavities with MZIs in a tapered optical fiber to sensing temperature and strain to a certain amount. They can accomplish simultaneous measurement of two parameters. However, the length of MZI is typically longer than necessary, which is harmful to the compactness of the design. However, the size of MZI is sometimes considerable, which detracts from its compactness.

### 4.4. Based on Photonic Crystals Fiber (PCF)

**Figure 6:**
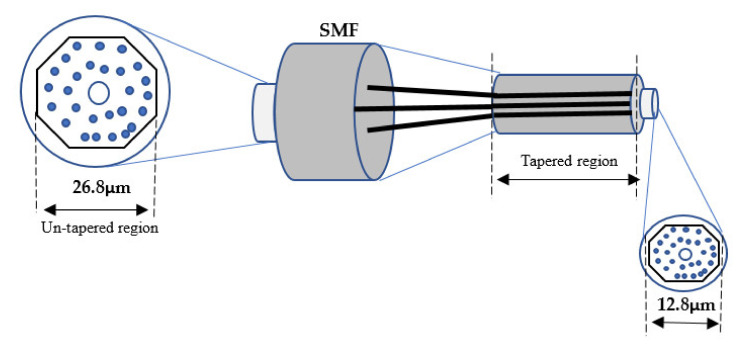
Tapered fiber based on photonic crystals fiber (PCF) [94].

Initially, PCF was named holes fiber due to air holes in its construction, also known as microstructure fiber, and PCF has two categories: index guiding and photonic band gap. Materials having a photonic band gap (PBG) have features that give them useful next-generation photonic device applications [92,93] and can be used to improve a temperature-independent MZI-based refractometer. This refractometer was built by sandwiching a 29 mm long, tapered PCF between two standard SMFs. The PCF’s air holes completely closed in spliced areas of fusion. This shows that PCF tapering enhanced the sensitivity of the refractometer [94,95,96], a RI range of 1.3355 to 1.4130, and a sensitivity of 1529 nm/RIU. Due to the PCF’s extremely low-temperature sensitivity, it was claimed to have a sensitivity of 1600 nm/RIU, approximately eight times that of interferometer not tapered with PCF [97], and it suggested a gas sensor using a nano-beam photonic crystal cavity connected to a tapered optical fiber. The method used seven pairs of tapered air holes, and ten pairs of mirror holes are the nano-beam cavities. A shift in the RI of the gas leads to a linear change in the resonance wavelength, with a sensitivity attributed to the gas of around 0.19 nm/10−3 RIU [98]. The construction of photonic crystals with a linearly tapered waveguide is suggested to gradually vary the waveguide width. Four drop channels are supplied to enable applications such as wavelength de-multiplexing, and waveguide has been tapered to confine the propagating light to the required locations [99]. Figure 6 shows tapered fiber based on photonic crystals fiber (PCF).

### 4.5. Based on Surface Plasmon Resonance (SPR)

**Figure 7:**
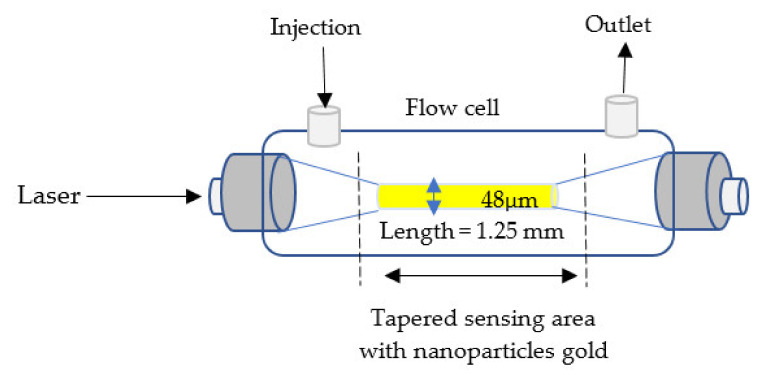
Tapered fiber optic based on surface plasmonic resonance (SPR) [105].

Surface plasmon resonance (SPR) is a very effective instrument widely utilized in chemical, biological, and medical applications. It is a type of refractometric sensing device that measures refractive index (RI) changes in the field. The coupling medium via the contact is required for photon energy excitation. It can do so by utilizing the optical system’s form structures, such as prism coupling (a method known as the Kretschmann design), localized surface plasmon (LSPR), waveguide, fiber-optic, and grating structures [100,101,102,103,104]. Equation (14) illustrates the resonance condition required for SPR:

(14) $$\sqrt{\varepsilon_{p}}\;\sin\theta_{res}=\sqrt{\frac{\varepsilon_{m}\;\varepsilon_{d}}{\varepsilon_{m}+\varepsilon_{d}}}$$

where $\varepsilon_{p}$, $\varepsilon_{m}$, and $\varepsilon_{d}$ denote the dielectric constants of the substrate (prism, optical fiber backbone, etc.), a plasmonic material (metals), and a dielectric layer (analyte medium), respectively, and $\theta_{res}$ denotes the incident resonance angle. The optical tapered fiber-based SPR configuration where a taper is coated with a thin metal film has several advantages over the standard SPR configuration, including its small size, light weight, and automatic alignment sacrificing sensitivity or overall sensor performance. Label-free surface plasmon resonance (SPR) sensor based on a tapered fiber is used in biochemical diagnosis. The sensing method examines the change in transmission intensity caused by deposited gold nanoparticles on the tapered fiber surface owing to evanescent field absorption [105,106], as shown in Figure 7.

### 4.6. Based on Multi-Taper Devices

**Figure 8:**
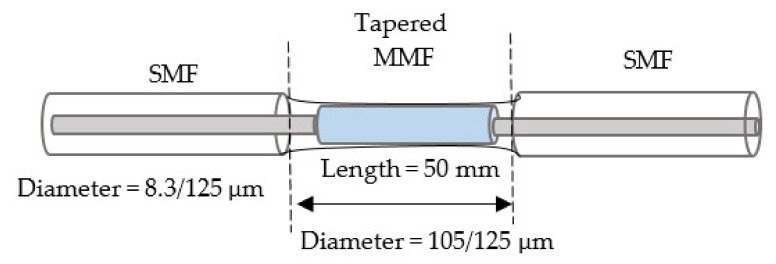
Tapered fiber optic based on multi-taper devices [108].

In this section, a multi-mode interference (MMI) device has been thoroughly described in a planar waveguide. Typically, these properties become useful at self-imaging of the light input field, a well-established technique commonly used in the design of optical communications beam splitters, combiners, and multiplexers [107,108]. The authors suggested a refractive index (RI) sensor structure based on multi-tapered by using single-mode/multi-mode fibers. The tapered area generates the evanescent wave, which penetrates the surrounding liquid. The methodology used was manufacturing three, five, and eight tapers, and the results were RI sensitivity of 261.9 nm/RIU in the RI range of 1.3333–1.3737 [109]. In another report, the fiber refractometer is illustrated using MMF sandwiched between two SMF half-tapers. At an external RI of 1.333, the produced interference fringe moves toward longer wavelengths with a 319.3 nm/RIU response sensitivity. The response sensitivity increases monotonically to 500.6 nm/RIU as the RI operates from 1.333 to 1.411 [110]. The researchers proposed a new intensity-modulated RI sensor based on a single-mode-single-mode (FT-SMS) fiber structure. The front taper is produced using a flame-heated technique on MMF. The beam propagation method is utilized to investigate the taper region intensity feature, and comprehensive RI and temperature testing are then performed. According to the experimental results, the sensitivity is 342.815 dB/RIU, between 1.33 and 1.37. The equivalent resolution is $2.92\times 10^{5}$ RIU, about four times that of wavelength demodulation [111], as shown in Figure 8.

### 4.7. Based on Fiber Loop Ring-Down (FLRD) Technology

**Figure 9:**
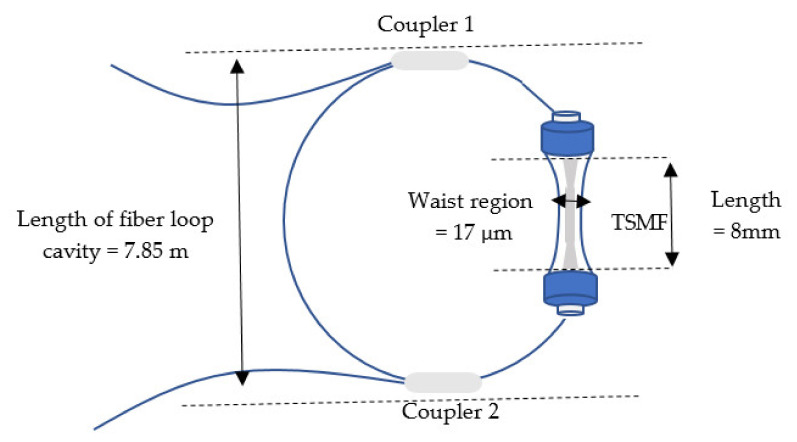
Tapered fiber optic based on fiber loop ring-down (FLRD) [115].

FLRD is a sensitive absorption method in which the rate of absorption in an optical cavity is measured. The effective absorption path length is very long. In addition, the sensitivity independent of the intensity of light source fluctuations [112]. A study of a constructed RI sensor based on tapered fiber loop ring-down (FLRD) was described, and the ring-down contributed to time changes with the intensity loss near the tapered SMF. Results indicated a sensitivity of 388.581 RIU and a detection limit of less than $2.57\times 10^{5}$ RIU [113]. A fiber-optic sensor with an S fiber taper (SFT) structure is presented. The SFT is used in line with MZI and is placed into a compensating fiber loop for the aim of evaluating the RI by improving the fiber loop properties. The surrounding refractive index may be readily determined by querying the fiber loop’s ring-down time. Results show a sensitivity of 3128.954 μs/RIU during a range of 1.3330–1.3682 [114]. A new refractive index sensing technique based on the chaotic association FLRD method is suggested. The results show that the sensitivity improves as an inverse relationship with a decrease in the loop length. Furthermore, the loop length has a minor effect on the system detection limit of $10^{4}$ RIU [115], as shown in Figure 9.

### 4.8. Based on Optical Tweezers

**Figure 10:**
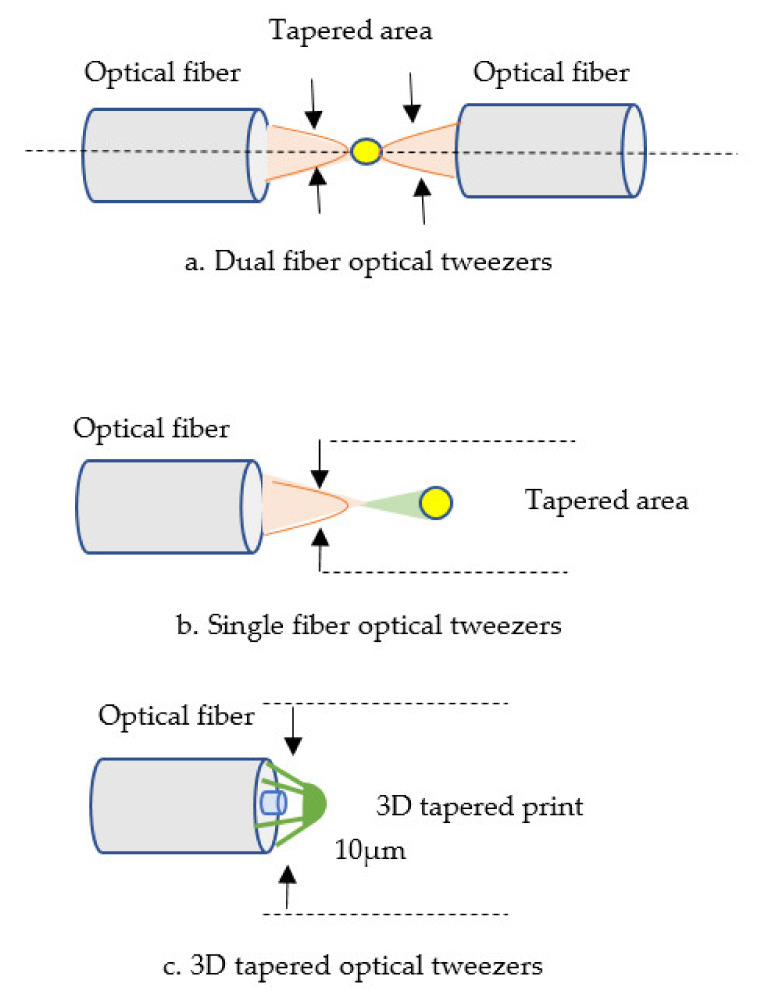
Tapered fiber optic based on optical tweezer structures [122].

Optical tweezer tools are commonly applied for many fields of application, such as biological, genetic, neurosciences, molecular medicine, pharmacy, and biotechnology. They are used for manipulating microscopic objectives, such as cells and bacteria, by focusing light beams (intensity maximum occurs) on nanoparticles. Due to their ease of manipulation, narrow shape, and ease of manufacture, optical fiber tweezers have garnered substantial attention in optical trapping in recent years [116,117,118]. A pair of SMF tweezers for biomedical use and multi-trapping research was presented. The trapping force feature of this pair of SMF tweezers is computed using the finite time-domain method. Results demonstrate that the number of particles trapped is related to refractive index and particle size [119,120,121]. Combining 3D-printed Fresnel lenses with a dual fiber surface is a good approach for increasing trapping efficiency and stability in the optical tweezer method. In dual-fiber traps, the use of a converging beam rather than a diverging beam results in a high trapping efficiency in both the axial and transverse directions. Diffractive Fresnel lenses connect beams with a numerical aperture of up to 0.7. These lenses give a long focus distance of up to 200 m in a medium with a relatively high refractive index. Femtosecond two-photon lithography enables the fabrication of such diffractive lenses with microscale features at the fiber tip [122], as shown in Figure 10.

**Table 1:** Studies analysis for categorizes tapered-fiber forms.

Tapered Method	Taper Length	Sensitivity	RI Scale OR Wavelength Scale	Waist Diameter	Publishing		Application	Ref
					Quantity	Quality		
FBG	100 μm	0.90, −38.49 pm/MPa 12.03 pm/°C	0.7250, 0.9980	30 μm	✓	✓	Temperature and pressure	[64]
FBG	10 mm	1129.44 pm/με −54.58 pm/°C	NA	90 μm	✓	✓	Temperature and strain	[70]
FBG	800 μm 750 μm	459.974 nm/RIU 420.781 nm/RIU	1.3540–1.3810	60 μm	×	✓	Biological, medical, and chemical	[63]
FBG	780 μm	269.76 dB/RIU	1.3330–1.4060	54 μm	×	✓	Temperature	[62]
LPFG	619.24 μm	45.87 pm/°C −52.57 nm/RIU	1.33–1.37	44.81 μm	×	✓	Temperature	[73]
LPFG	730 μm	8188 nm/RIU	1.33–1.34	12 μm	×	✓	Biological and chemical	[75]
MZI	∼35.7 μm	∼8239 pm/MPa 0.0055 MPa/°C	1.3241–1.3280	24 μm	✓	✓	Temperature and pressure	[82]
MZI	350 μm 200 μm	28 nm/vol	1.3645	55 μm	×	✓	Ethanol concentration	[87]
MZI	625 mm	213.235–215.294 nm/RIU 0.089 and 0.094 nm/°C	0.089	65 mm	×	×	Temperature	[90]
LPFG	2.3 mm 2.5 mm	1.82 pm/με–8.17 pm/με 47.9 pm/°C and 65 pm/°C	Wavelength shifting 1539.4 nm to 1541.2 nm,	62.5 mm	×	×	Temperature and strain	[76]
MZI	4 mm	∼4202 nm/RIU 41 pm/°C	1.3241–1.3280	95 μm 60 μm	✓	✓	Temperature	[85]
SPR	1.25 mm	3.2 × 10^5^ RIU	1.333–1.403	48 μm	×	✓	Biochemical and biomolecular	[105]
SPR	25 mm	18 nm/RIU	1.3324–1.4254	15 μm	×	×	Biological and chemical	[106]
PCF	∼2 cm	1600 nm/RIU	1.3333–1.3577	61 μm top 49 μm mid 30 μm bottom	✓	✓	Biochemical and biomolecular	[96]
PCF	29 mm	1529 nm/RIU	1.3355–1.413	71.7 μm	✓	✓	Environments, biomolecules	[97]
Multi- devices	550 μm	261.9 nm/RIU	1.3333–1.3737	52 μm	✓	✓	Biological and chemical	[109]
Multi- devices	17.8 mm	−342.815 dB/RIU	1.33–1.37	29.2 μm	✓	✓	Biochemical and environments	[111]
Loop ring-down	8 mm	0.045 ns^−1^RIU^−1^	1.3347–1.3721	17 μm	✓	✓	Medical pharmaceuticals, industrial fluids, photochemical plastics, and food industry	[115]
Loop ring-down	795 μm	−3128.954 μs/RIU	3330–1.3682	65 μm	✓	✓	Biochemistry	[114]
Loop ring-down	782 μm	−388.581 μs/RIU	1.335–1.375	28 μm	✓	✓	Industrial processing and bio/chemical	[113]
Multi- devices	3 mm	500.6 nm/RIU 319.3 nm/RIU	1.333 to 1.411	17 μm	✓	✓	Chemical and biological	[110]
Optical tweezer	200 μm 380 μm	NA	NA	1 μm 3.3 μm	✓	✓	Particle and single-cell microscopy	[122]
Optical tweezer	20 μm	NA	1.33–1.40	2.5 μm 3.7 μm	×	×	Biomedical	[119]

## 5. Opportunities and Challenges

One of the main problems for future applications of tapered optical fiber sensors is manufacturing the devices, which must be dependable and reproducible. As we know, taper devices are not selectively sensitive in their most basic form and require specificity-enhancing coating. Furthermore, there are difficulties in handling such as packing and mechanical strength. In addition, the limit of diffraction is the essential restriction to trapping. It is difficult to confine particles inside the diffraction limit, with limited distance around the trapped particle. The limit of diffraction also makes it challenging to combine additional manipulation and measurement activities that must be concurrent [123] and to achieve excellent symmetry while avoiding the volatility of the waist diameter induced by flame turbulence [124,125,126,127]. Due to the tiny size of the fiber core, the communication fiber connector demands extreme accuracy. The influence of lateral displacement on the coupling effectiveness of fiber connectors was reported. The innovative directional tapered description optical fiber connection is then constructed, considering that optical fiber is often used in pairs. One is utilized for transmission and the other for reception. The direction of signal transmission tapers the redesigned connector fiber head construction [128,129]. Moreover, the future opportunities to advance (micro/nano) techniques and develop fibers with unique optical characteristics require little question that designs and fabrications of innovative microstructured optical sensors will continue to be a growing research field. The proposed development of microstructured fiber optic sensors will face both challenges and possibilities [41], for example, optical fiber potential sensing selectivity with the dual-parameter measuring capability to date. However, there are requirements to reduce the error of multi-parameter measurements and expand the dual-parameter size to three-parameter or over. In addition, there is higher sensitivity because more accurate measurements in chemical and biological sectors single-molecule identification and gas sensing are desired. Chemical and biological single molecule identification and gas sensing can be an intelligent and flexible sensor design with varied target samples and different conditions. Long stability and the robustness of the small structures are critical to sensing effectiveness and longevity devices. Finally, optical fiber has many essential features such as flexible input/output, capability, and light delivery for remote instruments. Consequently, optical tapering might apply for on-site analytics and portable mini-spectrometers. Table 2 summarizes the challenges of tapered fiber structures.

## 6. Conclusions

In summary, this narrative review evaluated many types of common tapered fiber structures in different optical sensors forms and their respective concepts and applications, besides their theoretical, manufacturing techniques, and features. Furthermore, tapered fiber could be designed as sensors for various measurands, including sensors for RI, temperature, humidity, and bio-sensing, have been considered. In addition, this review also assesses the effects of the length and waist optical taper for improving the sensitivity. As a result, they provide more freedom in producing focusers with unique spot sizes. Therefore, tapered fiber can be potentially the most effective in medicinal and environmental applications. Finally, this analysis provides a road map of fundamental scientific primer for better understanding on the difficulties and implementation to improve optical sensors in this field.

## Figures and Tables

**Table 2:** Shows the challenges of tapered fiber techniques.

Tapered Method	Challenges
Fiber Bragg grating (FBG)	• Some spectra of reflection overlapped. • Unstable interference wave. • Complex configuration for grating inscription. • Insufficient sensitivity.
Long-period fiber grating (LPFG)	• The spectrum is limiting. • Cross-sensitivity problem. • The roughness of the surface.
Mach–Zehnder (MZI)	• Spectral band-width is limited. • Manufacturing cost. • Sensitivity enhancement is required.
Photonic crystals (PC)	• The samples given are limited. • The manufacturing of metal layers is a complex procedure. • Integration is expensive. • Mechanical reliability is weak, and mass production is challenging.
Surface plasmon resonance (SPR)	• The life of the sensitive layer controls the lifetime of the device. • Small sample size. • There is a need for practical application, including different sample collection processes. • The waist area is rather hard.
Multi-taper devices	• Measured refractive index range is limited. • Limited stability. • The roughness of the surface.
Fiber loop ring-down technology	• The samples given are limited. • Measuring accuracy depends on the high wavelength resolution of the de-modulation device.
Optical tweezers	• Obtain an analytical formula for the output light field is difficult. • The trapping of optical tweezers is inadequate due to the limit of laser beam diffraction. • Dramatic disruptions from molecular diffusion.

## Data Availability

Not applicable.
